# Formyl-peptide receptor 2 signalling triggers aerobic metabolism of glucose through Nox2-dependent modulation of pyruvate dehydrogenase activity

**DOI:** 10.1098/rsob.230336

**Published:** 2023-10-25

**Authors:** Tiziana Pecchillo Cimmino, Ester Pagano, Mariano Stornaiuolo, Gabriella Esposito, Rosario Ammendola, Fabio Cattaneo

**Affiliations:** ^1^ Department of Molecular Medicine and Medical Biotechnology, School of Medicine, University of Naples Federico II, 80131 Naples, Italy; ^2^ Department of Pharmacy, School of Medicine, University of Naples Federico II, 80131 Naples, Italy

**Keywords:** formyl peptide receptors, NADPH oxidase, reactive oxygen species, tyrosine kinase receptor transactivation, glucose metabolism, Warburg effect

## Abstract

The human formyl-peptide receptor 2 (FPR2) is activated by an array of ligands. By phospho-proteomic analysis we proved that FPR2 stimulation induces redox-regulated phosphorylation of many proteins involved in cellular metabolic processes. In this study, we investigated metabolic pathways activated in FPR2-stimulated CaLu-6 cells. The results showed an increased concentration of metabolites involved in glucose metabolism, and an enhanced uptake of glucose mediated by GLUT4, the insulin-regulated member of GLUT family. Accordingly, we observed that FPR2 transactivated IGF-IR*β*/IR*β* through a molecular mechanism that requires Nox2 activity. Since cancer cells support their metabolism via glycolysis, we analysed glucose oxidation and proved that FPR2 signalling promoted kinase activity of the bifunctional enzyme PFKFB2 through FGFR1/FRS2- and Akt-dependent phosphorylation. Furthermore, FPR2 stimulation induced IGF-IR*β*/IR*β*-, PI3K/Akt- and Nox-dependent inhibition of pyruvate dehydrogenase activity, thus preventing the entry of pyruvate in the tricarboxylic acid cycle. Consequently, we observed an enhanced FGFR-dependent lactate dehydrogenase (LDH) activity and lactate production in FPR2-stimulated cells. As LDH expression is transcriptionally regulated by c-Myc and HIF-1, we demonstrated that FPR2 signalling promoted c-Myc phosphorylation and Nox-dependent HIF-1*α* stabilization. These results strongly indicate that FPR2-dependent signalling can be explored as a new therapeutic target in treatment of human cancers.

## Graphical abstract

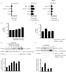

## 1. Introduction

G protein-coupled receptors (GPCRs) and tyrosine kinase receptors (TKRs) play critical roles in health and disease and represent the major classes of cell surface receptors. GPCRs bind a structurally diverse range of ligands [1] which trigger downstream signalling via heterotrimeric G protein dissociation (G*α* and G*βγ* subunits) [2]. TKRs bind growth factors which typically induce dimerization of receptor monomers triggering trans-autophosphorylation of COOH-terminal tyrosine residues that act as recruitment sites for intracellular adaptor proteins. Typically, TKR-mediated signalling is a driver for cell proliferation, migration and survival.

GPCR-mediated TKR transactivation represents a molecular mechanism necessary to increase the number and range of cellular signalling networks, by integrating the diversity of GPCRs and their ligands with the large signalling networks related to TKRs [3]. Trans-phosphorylation has been implicated in physiological and pathophysiological processes and has been observed for several receptor pairings in many cell types [4,5]. As involved molecular mechanisms and signalling effectors can vary with receptor couple [6], the GPCR/TKR interactions may be considered attractive new targets for drug discovery programmes.

Stimulation of several GPCR induces a low increase of NADPH oxidase (Nox)-dependent reactive oxygen species (ROS) concentration, that act as signalling molecules in several cellular processes, such as phosphorylation of kinases, activation of transcription factors and TKR transactivation [7–14]. The classical NADPH oxidase of phagocytes consists of five subunits: p67^phox^, p47^phox^, p40^phox^, p22^phox^ and the catalytic subunit gp91^phox^. Members of this family, identified in several nonphagocytic cells, are homologues of the catalytic subunit gp91^phox^ and are named Nox1, Nox3, Nox4, Nox5, Duox1 and Duox2. Nox2 is also known as gp91^phox^. Nox activity is controlled by p47^phox^ and p67^phox^ regulatory subunits, their homologues NOXO1 and NOXA1, or DUOXA1 and 2. Moreover, the GTPase Rac modulates the activity of several of these enzymes [15]. Nox1, Nox2, Nox3 and Nox5 are transmembrane proteins that transport electrons across biological membranes to reduce oxygen to superoxide. Nox4, Duox1 and Duox2 do not produce superoxide, but hydrogen peroxide [7–14]. Members of the Nox family have been identified as the major sources of ROS generation in cancer cells [16] and, among these, Nox2 is strongly expressed in several epithelial cancer cells, such as lung [17], ovarian [18], breast [19], cervical [20] and prostate cells [21]. ROS that are generated by Nox enzymes in non-phagocytic tissues are well documented second messengers in a variety of signalling pathways in several cell types [22]. Molecular mechanisms through which ROS modulate cell signalling depend on their capacity to oxidize cysteine residues within proteins, which can function as redox sensors and transducers of ROS-primed signalling [23]. Therefore, cells can sense ROS to variable levels through the reversible oxidation of cysteine residues allowing a gradual response to intracellular ROS concentrations.

The human formyl-peptide receptor (FPR) family is clustered on chromosome 19 and encodes three Class A GPCRs involved in neutrophil chemotaxis and in innate immune responses, through recognition of pathogen-associated molecular patterns (PAMPs) and damage-associated molecular patterns (DAMPs) [24]. FPR2, a member of this family, is highly expressed in myeloid cells and in cells of diverse origin [25], as well as on the nuclear membrane of CaLu-6 and AGS cells [26]. FPR2 is activated by an array of ligands including proteins, peptides and lipids. Most of them, besides inducing chemotaxis, also stimulate pro-inflammatory processes, pro-resolving or anti-inflammatory pathways [27], depending on the nature of the agonist and on the different receptor domains they used [28,29]. The switch between pro-inflammatory and anti-inflammatory responses is due to conformational changes of FPR2 upon ligand binding [29]. The peptide WKYMVm, annexin A1 (ANXA1) and lipoxin A4 (LXA4) are well-known anti-inflammatory FPR2 ligands [30–32]. On the other hand, serum-amyloid alpha (SAA) and *β*-amyloid act as pro-inflammatory agonists on FPR2 [33]. FPR2 contributes to detrimental effects in cancer progression. In fact, invasion of ovarian cancer cells requires FPR2 activation by the cathelicidin LL-37 [34], the Hp(2-20) peptide, that efficiently binds FPR2, promotes the migration and proliferation of gastric cancer cells [35] and ANXA1 stimulates the development and progression of astrocytoma [36]. However, the role of FPR2 in cancer progression is still controversial and seems related to the nature of its ligands and of cell type, as demonstrated by the observation that LXA4 attenuates pancreatic cell invasion [37].

FPR2 stimulation triggers the activation of several protein kinases and, in turn, the phosphorylation of several cytosolic signalling proteins [13,25,38] involved in the modulation of proliferation, differentiation, migration, communication, and other critical intracellular functions [39]. FPR2-dependent phosphorylated molecules include also non-signalling proteins, such as the cytosolic subunits p47^phox^ and p67^phox^ of NADPH oxidase, whose phosphorylation is required for the full activity of the NADPH oxidase complex [12,14].

Protein kinases mediate a network of highly complex signals. Many proteins, including TKRs [3], are phosphorylated and the main mechanism of regulation is represented by the switch ‘phosphorylation/dephosphorylation’, in which protein phosphatases (PTPases), through the reversible oxidative inhibition of reactive cysteine residues, play a crucial role [40–42]. We previously demonstrated that FPR1 and FPR2 stimulation induces ROS-dependent TKR transactivation, as well as the phosphorylation and nuclear translocation of regulatory transcriptional factors [9–11,14,43]. Protein kinases and PTPases act synergistically and their impaired regulation or activation is responsible of several human diseases. Multiple phospho-sites, identified in both protein kinases and phosphatases, contribute decisively to expand the repertory of molecular mechanisms of regulation or for fine-tuning of switch properties [44].

By using a phospho-proteomic approach we previously demonstrated that FPR2 stimulation induces redox-regulated phosphorylation of numerous proteins [38,44]. We classified FPR2-dependent phosphorylated proteins according to their known or putative functions and this analysis revealed that most of them participated in metabolic processes. About 33% of the proteins of this group is involved in biosynthetic processes and the remaining 67% of proteins is involved in cellular metabolic processes, including primary metabolism [38]. We also demonstrated that the binding of specific FPR2 agonists enhances the non-oxidative phase of pentose phosphate pathway (PPP), improves the expression of the ASCT2 glutamine transporter and induces the de novo synthesis of pyrimidine nucleotides [45].

Herein, we apply a metabolomic approach to analyze the metabolic pathways activated in human CaLu-6 epithelial carcinoma cell line, following stimulation of FPR2 with the WKYMVm peptide or ANXA1. Obtained results prove that the agonist-mediated stimulation of the receptor triggers intracellular redox signalling pathways involved in glucose uptake and aerobic metabolism of glucose typical of the Warburg effect.

## 2. Material and methods

### 2.1. Cell culture and reagents

CaLu-6, A549 (ATTC, Manassas, VA, USA) and p22phox^Crispr/Cas9^ CaLu-6 cells were cultured in Dulbecco's modified Eagle's medium (DMEM) supplied with 10% fetal bovine serum (FBS) (Invitrogen Corp., Carlsbad, CA, USA) at 37°C and 5% CO_2_. Cells were grown to 70% confluence, serum starved for 24 h and stimulated or not with 10 µM WKYMVm (Primm, Milan, Italy) or 10 nM ANXA1 for various times, as indicated in the figures. CaLu-6 cells were also preincubated with WRWWWW (WRW4) (Primm, Milan, Italy) for 15 min at a final concentration of 10 µM, or with apocynin (Sigma Chemical, St Louis, MO, USA) for 2 h at a final concentration of 5 mM, or with PP2 or with PP3 (Calbiochem, La Jolla, CA, USA) for 45 min at the final concentration of 10 µM, or with GSK1904529A (MedChemExpress, Monmouth Junction, NJ, USA) for 2 h at the final concentration of 3 µM, or with LY2874455 (MedChemExpress, Monmouth Junction, NJ, USA) for 2 h at the final concentration of 5 µM, or with AG1478 (Calbiochem, La Jolla, CA, USA) for 60 min at the final concentration of 10 µM, or with wortmannin (Calbiochem, La Jolla, CA, USA) for 60 min at the final concentration of 0.5 µM, or with LY294002 (Calbiochem, La Jolla, CA, USA) for 60 min at the final concentration of 10 µM, before stimulation with 10 µM WKYMVm or 10 nM ANXA1.

### 2.2. p22phox^crispr/Cas9^ double-nickase CaLu-6 cells

p22phox^Crispr/Cas9^ cells were generated by transfecting CaLu-6 cells with Double Nickase Plasmid or with a Double Nickase Plasmid control (Santa Cruz Biotechnology, Irvine, CA, USA) following the manufacturer's instructions, as previously described [25]. Positive selection of CaLu-6-transfected cells was performed in medium containing puromycin for 5 days. Single clones were isolated, cultured separately, and tested by western blotting to analyze p22^phox^ expression (data not shown). p22^phox^ knockout clones were collected in order to obtain p22phox^Crispr/Cas9^ CaLu-6 cells.

### 2.3. Metabolomic analysis by liquid chromatography–mass spectrometry

Metabolomic analysis by LC-MS was performed in growing and in 24 h serum starved CaLu-6 cells stimulated or not with WKYMVm in presence or absence of WRW4. Briefly, 2 × 10^4^ cells were plated in 48-multiwell plate and the day after were serum-starved for 24 h before the treatments. Cell monolayers were rinsed in cold water and then lysed in 400 µl of a 1:1 prechilled MetOH:H_2_O solution. The samples were vortex-mixed, kept on ice for 20 min, and centrifuged again at 10 000 × *g*, at 4°C for 10 min. The collected supernatant was dried in a SpeedVac concentrator system (Thermo Scientific), operated at room temperature. Dried supernatants were reconstituted with 125 µl of methanol/acetonitrile/water (50:25:25). Extracted metabolites were analysed using an ACQUITY UPLC system online coupled to a Synapt G2-Si QTOF-MS (Waters Corporation, Milford, MA, USA) in positive and negative modes in the following settings: reverse-phase ACQUITY UPLC CSH C18 (1.7 µm, 100 × 2.1 mm^2^) column (Waters), 0.3 ml min^−1^ flow rate, mobile phases composed of acetonitrile/H_2_O (60 : 40) containing 0.1% formic acid and 10 mM ammonium formate (phase A), and isopropanol/acetonitrile (90 : 10) containing 0.1% formic acid and 10 mM ammonium formate (phase B). Peak detection, metabolite identification and quantitation were performed as previously described [46], fitting experimental data with internal standard and calibration curves. Data analysis was conducted and heatmaps were generated with the on-line software MetaboAnalyst (https://www.metaboanalyst.ca), as previously reported [47,48] (electronic supplementary material, table S1).

### 2.4. 2-NBDG glucose uptake assay on CaLu-6 cells

CaLu-6 cells were plated (5 × 10^3^ per well) in a black, clear bottom, 96-well microtiter plate (Perkin Elmer, Waltham, USA) in a final volume of 100 µl per well of culture medium. After 24 h the culture medium was carefully removed and replaced with 100 µl of HBSS containing 100 µM 2-deoxyglucose (2-DG), 0.4 g l^−1^ BSA, and 1.3 mM CaCl_2_ (in the absence of any growth factors or FBS) and were incubated with WKYMVm at the final concentration of 10 µM for the indicated times in presence or absence of WRW4. Plates were incubated at 37°C for 1 h. Treatments were performed in triplicate and the results are the mean of three independent experiments. Medium was replaced with the same HBSS supplemented with 100 µM 2-DG and 6 µM 2-(*N*-(7-nitrobenz-2-oxa-1,3-diazol-4-yl)amino)-2-deoxyglucose (2-NBDG). Plates were incubated with the fluorescent probe for 45 min and then washed twice in PBS. Uptake of 2-NDBG was measured in a Perkin Elmer Envision 2105 multiplate reader (Perkin Elmer), using the inbuilt monochromator and the following parameters: *λ* excitation 471 nm, *λ* emission 529 nm, and monochromator cut off 360 nm. After the measurement of 2-NDBG, cells were fixed in 3.7% paraformaldehyde for 30 min to be then permeabilized in 0.1% Triton X-100 in PBS and stained with the nuclear dye DAPI (30 µM). This second fluorescence measurement correlates with the total number of cells in each well and was used for normalization. DAPI fluorescence was measured using the following parameters: *λ* excitation 351 nm and *λ* emission 450 nm. Data analysis for glucose uptake is reported as the ratio between intracellular 2-NDBG fluorescence and DAPI fluorescence ± s.d.

### 2.5. Protein extraction and western blot

Proteins were purified from 24 h serum-starved CaLu-6 or p22phox^Crispr/Cas9^ CaLu-6 cells stimulated or not with 10 µM WKYMVm, in the presence or absence of selective inhibitors, as described above. Whole lysates were obtained by scraping cells with ice cold RIPA buffer (50 mM Tris–HCl, pH 7.4, 150 mM NaCl, 1% NP-40, 1 mM EDTA, 0.25% sodium deoxycholate, 1 mM NaF, 10 µM Na_3_VO_4_, 1 mM phenyl-methyl-sulfonyl-fluoride, 10 µg ml^−1^ aprotinin, 10 µg ml^−1^ pepstatin, 10 µg ml^−1^ leupeptin), as previously described [49].

Membrane lysates were purified as mentioned above [26]. Cells were lysed in hypotonic buffer containing 10 mM Tris–HCl, 1 mM CaCl_2_, 150 mM NaCl, 1 mM phenyl-methyl-sulfonyl-fluoride, and a protease inhibitor cocktail (10 µg ml^−1^ aprotinin, 10 µg ml^−1^ pepstatin, and 10 µg ml^−1^ leupeptin) (Buffer II) and centrifuged at 400 × *g* for 10 min at 4°C, in order to obtain a cytosolic and a membrane fraction. Membrane fraction was incubated overnight at 4°C in constant agitation with a buffer containing 125 mM Tris–HCl, 1 mM phenyl-methyl-sulfonyl-fluoride, 1% Triton X100, and the protease inhibitor cocktail (Buffer II).

Bio-Rad protein assay was used to determine protein concentrations (BioRAD, Hercules, CA, USA). Western blot analysis on whole or membrane lysates was performed as previously described [50].

Anti-tubulin (SC-8035), anti-GAPDH (SC-47724), anti Na/K ATPase (SC-48345), anti-GLUT4 (SC-53566) and anti-phospho-c-Myc (S62) (SC-8000-R) antibodies were purchased from Santa Cruz Biotechnology (Irvine, CA, USA). Anti-phospho-IGF-IR (Y1131/1146), anti-phospho-PFKFB2 (S483), anti-phospho-FRS2 (Y436), anti-phospho-PDH (S293), anti-phospho-LDH (Y10), and anti-phospho-c-Src (Y416) were from Cell Signalling Technology (Denvers, MA, USA). Anti-HIF1*α* (NB100–105) was from Novus Biologicals (Centennial, CO, USA). Goat-anti-mouse (bs-0296G-HRP) and goat-anti-rabbit (bs-0295G-HRP) were from Bioss Antibodies (Woburn, MA, USA). Proteins were visualized by enhanced chemiluminescence reagent (Amersham Biosciences, Little Chalfont, Buckinghamshire, UK) and were quantified using densitometry (Chemidoc, Bio-Rad). Each experiment with relative densitometric quantification was separately repeated at least three times.

### 2.6. Lactate assay

Lactate concentration was measured in cell culture medium of CaLu-6 cells by Lactate-Glo Assay (Promega) following the manufacturer's instructions. Briefly, 1.5 × 10^4^ cells were seeded in 96-well plate. The day after, cells were serum-starved for 24 h, preincubated or not with 10 µM WRW4 for 15 min and then stimulated or not with 10 µM WKYMVm for 24 h. Five microlitres of medium was removed for each experimental point and diluted in 95 μl of PBS. For each experimental point, 50 µl of diluted medium was transferred to a 96-well assay plate and 50 µl of lactate detection reagent was added. Assay plate was shaken for 1 min to mix the reagents and incubated for 60 min at room temperature before recording luminescence. DMEM was used as a negative control. Luminescence was read with a Synergy H1 microplate reader (BioteK, VT, USA). Results are the mean of three independent experiments and, in each of these, every experimental point was analysed in triplicate.

### 2.7. Seahorse XF analysis

Extracellular acidification rate (ECAR) was measured by using the Seahorse XF Glycolytic Rate Assay Kit (Agilent, CA, USA). Calu-6 cells, cultured as described above, were seeded in the XF-24 cell culture plates at 20 000 cells per well, allowed to attach overnight and serum-starved for 24 h. Cells were incubated with 10 µM WKYMVm for 24 h followed by Seahorse assay. Then, medium was changed to Seahorse XF DMEM medium pH 7.4 (Agilent), supplemented with 25 mM glucose, 4 mM L-glutamine and 2 mM pyruvate, and allowed to equilibrate for 1 h in a CO_2_-free incubator at 37 °C. Real time measurement of ECAR was performed using an XF-24 Analyzer (Agilent).

### 2.8. Statistical analysis

Statistical analyses were evaluated by unpaired *t*-test to compare the mean of two independent groups of experiments or by one-way analysis of variance (ANOVA). GraphPad Prism 7 (GraphPad Software Inc., San Diego, CA, USA) was used to compare more than two experiments. All data reported are representative of at least three or more independent experiments and are expressed as means ± standard error mean (SEM). A *p* value of less than 0.05 was considered to be statistically significant.

## 3. Results

### 3.1. FPR2 stimulation induces glucose uptake and increases concentration of metabolites involved in glucose metabolism

**Figure 1.**
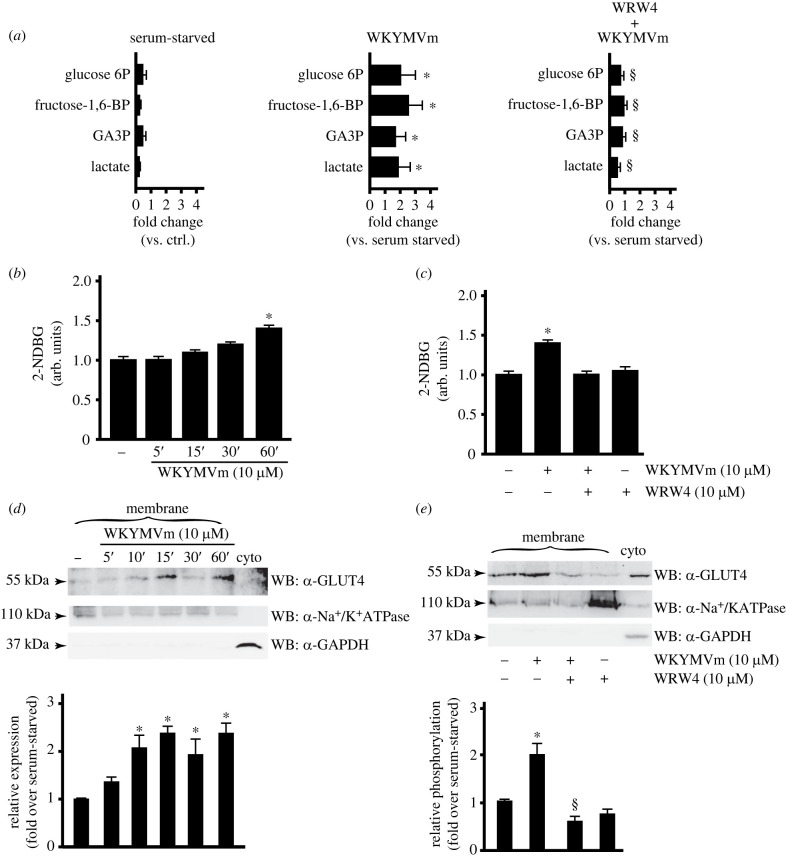
FPR2 stimulation enhances glucose metabolism. (*a*) FPR2-dependent modulation of metabolites involved in glucose metabolism. Growing cells (ctrl) were serum-starved for 24 h and then stimulated or not with 10 µM WKYMVm for 1 h in the presence or absence of 10 µM WRWWWW (WRW4). Metabolomic analysis was performed as described in Material and methods. (*b*,*c*) FPR2 stimulation induces glucose uptake. CaLu-6 cells were grown until reaching 80% of confluence, exposed to 100 µM 2-NDBG and incubated with WKYMVm at final concentration of 10 µM for the indicated times, in the presence or absence of WRW4. Uptake of 2-NDBG was measured in a Perkin Elmer Envision 2105 multiplate reader. Results are the mean of three independent experiments in which each point was analysed in triplicate. *p < 0.05 compared to unstimulated cells. (*d*,*e*) GLUT4 membrane translocation depends on FPR2 activation. CaLu-6 cells were serum-starved for 24 h and (*d*) stimulated for 5, 10, 15, 30 or 60 min with WKYMVm, or pretreated with (*e*) WRW4 before stimulation. Sixty micrograms of membranes lysates was immunoblotted with anti-GLUT4 antibody (α-GLUT4). Anti-Na^+^/K^+^ATPase antibody (α-Na^+^/K^+^ATPase) was used as a control for protein loading. Sixty micrograms of a cytosolic fraction (Cyto) was loaded and an anti-GAPDH antibody (α-GAPDH) was used as a control of cytosolic proteins. Data are representative of five independent experiments. *p < 0.05 compared to unstimulated cells. ^§^*p* < 0.05 compared to WKYMVm-stimulated cells. Glucose 6P: glucose 6-phosphate; fructose 1,6-BP: fructose 1,6-bis-phosphate; GA3P: glyceraldheide 3-phosphate.

We started profiling the metabolic response of CaLu-6 cells upon stimulation with WKYMVm. Compared to untreated cells, we observed an increased concentration of metabolites involved in glucose metabolism, such as glucose 6-phosphate, fructose 1,6-bis-phosphate (F1,6BP), glyceraldheide 3-phosphate (GA3P) and lactate (figure 1*a*). This increase was prevented by the preincubation with the FPR2 antagonist WRW4 (figure 1*a*), suggesting that FPR2 stimulation activated glucose oxidation via glycolysis. In this metabolic pathway, glucose is catabolized to pyruvate with production of 2 molecules of ATP and reduction of 2 mol of NAD^+^ to NADH per mole of glucose. Pyruvate, in aerobic conditions, is transported into mitochondria, where pyruvate dehydrogenase complex (PDC) catalyses its oxidative decarboxylation into acetyl-coenzyme A (CoA). This can feed the tricarboxylic acid (TCA) cycle and, in turn, the mitochondrial electron transport chain to produce energy. Pyruvate can be also reduced to lactate by a reaction catalysed by lactate dehydrogenase (LDH), and in our metabolomic analysis we interestingly observed that level of lactate increased in FPR2-stimulated cells (figure 1*a*). In cancer cells, this reaction defines the aerobic utilization of glucose typical of the Warburg effect [51].

Therefore, we evaluated the ability of the FPR2 agonist to stimulate glucose uptake in CaLu-6 cells. Treatment with 10 µM WKYMVm significantly increased glucose consumption in a time-dependent manner, when compared to control cells (figure 1*b*); this effect was prevented by pre-incubation with WRW4, before FPR2 stimulation (figure 1*c*). This result strongly suggests that WKYMVm-induced glucose uptake occurs through FPR2 activation. Enhanced glucose utilization is a known hallmark of cancer cells, which need glucose for energy production. Glucose uptake is mediated by members of transmembrane glucose transporter family, which include facilitative glucose transporters (GLUTs), sodium-glucose co-transporters (SGLTs), and transporters of the SWEET family, largely represented in plants [52]. The GLUT family includes 14 known transporters which are divided into three classes according to their structure. GLUT1 is upregulated in cancer by Src, Ras, Myc and Akt [53–56], and it is repressed by the tumor suppressor p53 [57]. GLUT4 is the insulin-regulated member of this family [58,59] and it is expressed in several cancer cells [60–62].

We analysed incorporation of GLUT1 and GLUT4 onto cell surface and observed that WKYMVm stimulation for different time spans induces GLUT4, but not GLUT1 (data not shown), membrane localization (figure 1*d*), which was prevented by WRW4 (figure 1*e*). In several experimental systems GLUT4 transport to the plasma membrane is regulated by the insulin-stimulated phospatidylinositol 3-kinase (PI3K)/Akt signalling pathway [63].

### 3.2. FPR2 signalling induces Nox2-dependent IGF-IR*β* and/or IR*β* transactivation

Intracellular signalling cascades triggered by FPR2 include the activation of several protein kinases, TKRs and PTPases [3,11,14,25,38,43,64]. As a result of FPR2-mediated TKR transactivation, cytosolic phospho-tyrosine residues of TKRs provide docking sites for recruitment and triggering of the STAT3, PLC-γ1/PKC*α* and PI3K/Akt pathways in different cell lines [11,14].

**Figure 2.**
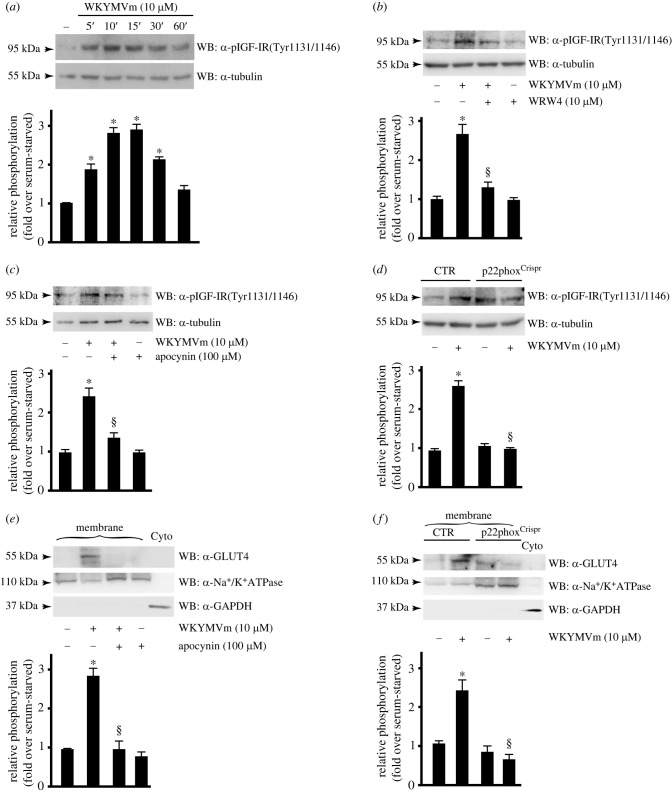
NADPH oxidase-dependent ROS generation modulates FPR2-mediated IGF-IR trans-phosphorylation and GLUT4 membrane translocation. (*a*–*d*) FPR2-dependent IGF-IR transactivation requires Nox2 activity. (*a*) CaLu-6 cells were growth-arrested for 24 h and stimulated for 5, 10, 15, 30 or 60 min with WKYMVm, or (*b*) pretreated with WRW4, or (*c*) preincubated with apocynin, before stimulation. (*d*) CaLu-6-control^Crispr/Cas9^ cells (CTR) and p22phox^Crispr/Cas9^ (p22phox^Crispr^) cells were serum-deprived for 24 h and then stimulated with WKYMVm. Fifty micrograms of whole lysates was resolved on 10% SDS-PAGE and incubated with an anti-pIGF-IR(Tyr1131/1146) antibody (α-pIGF-IR(Tyr1131/1146)). An anti-tubulin antibody (α-tubulin) was used as a control for protein loading. (*e*,*f*) GLUT4 membrane translocation depends on ROS generation. Sixty micrograms of membranes lysates was immunoblotted with anti-GLUT4 antibody (α-GLUT4). An anti-Na^+^/K^+^ATPase antibody (α-Na^+^/K^+^ATPase) was used as a control for protein loading. Sixty micrograms of a cytosolic proteins (Cyto) was loaded and an anti-GAPDH antibody (α-GAPDH) was used as a control. Data are representative of five independent experiments. *p < 0.05 compared to unstimulated cells. ^§^*p* < 0.05 compared to WKYMVm-stimulated cells.

Since GLUT4 is the insulin-regulated member of glucose transporter family, we analysed the ability of FPR2 to transactivate insulin-like growth factor-I receptor *β* (IGF-IR*β*) and/or insulin receptor *β* (IR*β*). Three tyrosine residues within the kinase domain (Y1131, Y1135 and Y1136) are the major autophosphorylation sites of IGF-IR*β* [65], which are necessary for kinase activation [66]. IR*β* shares significant structural and functional similarity with IGF-IR*β*, including the presence of an equivalent tyrosine cluster (Y1146, Y1150, Y1151). We used a monoclonal phospho-antibody able to detect both phosphorylated IGF-IR*β* and/or IR*β* and observed that FPR2 stimulation induces time-dependent IGF-IR*β* and/or IR*β* transactivation (figure 2*a*). Pre-treatment with WRW4, before WKYMVm stimulation, prevents IGF-IR*β*/IR*β* tyrosine phosphorylation (figure 2*b*), thus indicating that it depends on FPR2 activation.

In CaLu-6 cells FPR2 stimulation triggers Nox2 activation [14,25,38] and in several experimental systems the molecular mechanisms responsible for FPR2-dependent TKR trans-phosphorylation require Nox2 activity [11,14]. Therefore, we next preincubated CaLu-6 cells with apocynin, which prevents both p47^phox^ translocation and its interaction with p22^phox^ [67,68], before FPR2 stimulation and we observed that the pretreatment prevents IGF-IR*β*/IR*β* transactivation (figure 2*c*). By CRISPR/Cas9-based genome editing, we obtained a Calu-6 cell line expressing a non-functional form of p22^phox^ (p22phox^Crispr/Cas9^) [25]. Significantly, stimulation of these cells with WKYMVm failed to induce IGF-IR*β*/IR*β* phosphorylation (figure 2*d*), showing further evidences that ROS are signalling intermediates in TKR activation [69–72]. Since FPR2-mediated IGF-IR*β*/IR*β* transactivation depends on Nox2 activity (figure 2*c*,*d*) we investigated the role of Nox2 in GLUT4 membrane translocation. We preincubated Calu-6 cells with the Nox-specific inhibitor apocynin, before WKYMVm stimulation (figure 2*e*) and we incubated p22phox^Crispr/Cas9^ cells with the FPR2 agonist (figure 2*f*). The results show that blockade of Nox2 function prevents FPR2-induced GLUT4 translocation, suggesting that both FPR2-dependent glucose uptake and insulin receptor trans-phosphorylation are modulated by ROS generation.

### 3.3. FPR2 signalling induces glucose oxidation in the glycolytic pathway

Cancer cells increase glucose uptake and metabolism via glycolysis to meet the bioenergetic demands of rapid cell division [73]. Glycolysis is regulated at several steps via multiple mechanisms but the critical control point is the irreversible reaction catalysed by the 6-phosphofructo-1-kinase (PFK1) enzyme that converts fructose 6-phosphate (F6P) to F1,6BP. In our metabolomic analysis we observed an increase of F1,6BP in FPR2-stimulated cells (figure 1*a*). PFK1 is an allosteric enzyme regulated by fructose-2,6-bisphosphate (F2,6BP), the key activator of glycolysis, and by a variety of other metabolites. Intracellular F2,6BP levels are regulated by the bifunctional 6-phosphofructo-2-kinase/fructose-2,6-bisphosphatase (PFKFB2) enzyme that shows both kinase activity, which converts F6P to F2,6BP, and phosphatase activity, which catalyses the remotion of a phosphate in F2,6BP to generate F6P [74]. PFKFB exists as four isoenzymes (PFKFB1–4), the products of separate genes each with a distinct activity [74–76]. PFKFB2 is mainly expressed in lung, brain and heart [76,77], and its regulation by phosphorylation leads to an increase in F2,6BP concentration and thus to an enhanced glycolysis [78]. In human, the two main activating phosphorylation sites identified in PFKFB2 are Ser^466^ and Ser^483^ residues [78].

**Figure 3.**
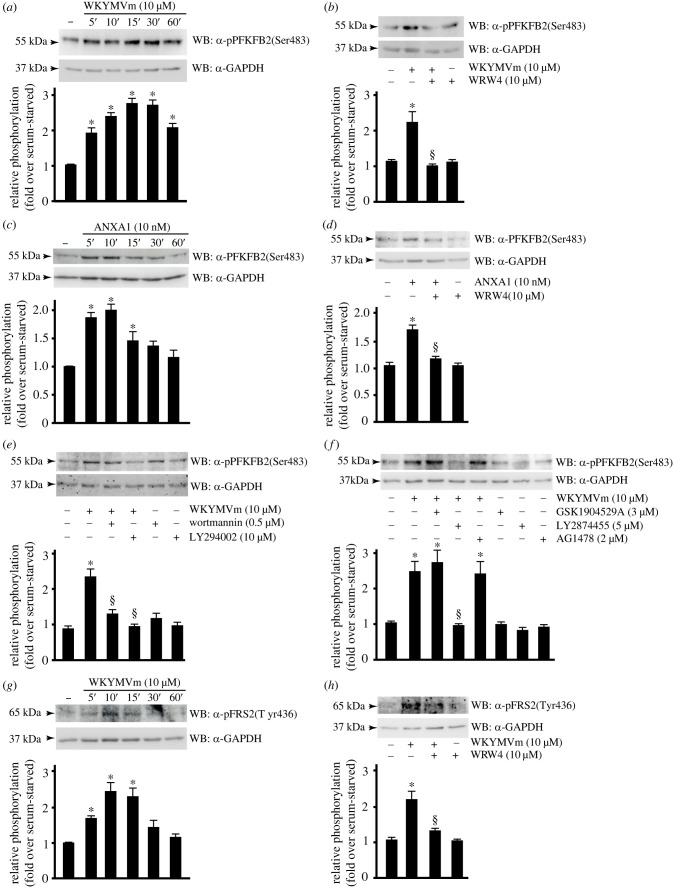
FPR2 signalling triggers FGFR1- and Akt-dependent glucose oxidation. (*a*–*d*) FPR2 stimulation induces PFKFB2 activation. (*a*) CaLu-6 cells were serum-deprived for 24 h and stimulated for 5, 10, 15, 30 or 60 min with WKYMVm or (*c*) with ANXA1. (*b*,*d*) Cells were preincubated with WRW4 before stimulation. (*e*–*h*) PFKFB2 phosphorylation depends on Akt activation and FGFR1 transactivation. (*e*) Cells were stimulated with 10 µM WKYMVm, or preincubated with wortmannin or LY294002, or (*f*) with GSK1904529A or LY2874455 or AG1478, before stimulation. (*g*,*h*) FPR2 signalling induces the activation of the scaffold phosphoprotein FRS2. (*g*) Serum-starved CaLu-6 cells were stimulated for increased times with WKYMVm as indicated, or (*h*) incubated with the FPR2 antagonist before stimulation. Fifty micrograms of whole lysates was resolved on 10% SDS-PAGE and immunoblotted with (*a*–*f*) an anti-pPFKFB2(Ser483) antibody (α-pPFKFB2(Ser483)), or with (*g*,*h*) an anti-pFSR2(Tyr436) antibody (α-pFSR2(Tyr436)). An anti-GAPDH antibody (α-GAPDH) was used as a control for protein loading. Data are representative of four independent experiments. *p < 0.05 compared to unstimulated cells. ^§^*p* < 0.05 compared to WKYMVm-stimulated cells.

We analysed FPR2-induced PFKFB2 phosphorylation by using an anti-phospho specific antibody and observed that either WKYMVm or ANXA1 trigger time-dependent PFKFB2 Ser^483^ phosphorylation (figure 3*a*,*c*), which was completely prevented by preincubation with WRW4 (figure 3*b*,*d*). Notably, the extent of PFKBP2 Ser^483^ phosphorylation appears to be sustained for longer times in cells stimulated with WKYMVm compared to ANXA1. Probably, these differences could be associated with the different nature of the agonists and thier different binding site in FPR2.

Since Ser^483^ residue of PFKFB2 is a target of Akt [78,79], we preincubated cells with wortmannin or LY294002 before WKYMVm stimulation and observed that these treatments prevented FPR2-induced PFKFB2 activation (figure 3*e*).

We analysed the role of other cell surface receptors, besides FPR2, involved on the activation of PI3K/Akt cascade and, in turn, in PFKFB2 Ser^483^ phosphorylation. To this aim we preincubated cells with GSK1904529A, that blocks IGF-IR autophosphorylation and downstream signalling [80], or LY2874455, a potent selective pan-FGFR inhibitor [81], or AG1478, a selective EGFR inhibitor [82], before WKYMVm stimulation. Western blot analysis showed that only FGFR inhibition prevents FPR2-induced PFKFB2 Ser^483^ phosphorylation (figure 3*f*), thus suggesting a cross-talk between FPR2 and FGFR in these cells. FGFR1–4 belong to the FGFR family of TKRs [83,84]. Ligand binding to FGFRs results in phosphorylation at Tyr^196^, Tyr^306^, Tyr^349^, Tyr^392^ and Tyr^436^ residues of the adaptor/scaffold phosphoprotein FGF receptor substrate 2 (FRS2) [85,86] and subsequent activation of PI3K/Akt pathway [87]. In immunoblot experiments we observed that WKYMVm stimulation induced a time-dependent phosphorylation of FRS2 at Tyr^436^ residue (figure 3*g*) which was prevented by the FPR2 antagonist (figure 3*h*).

These results demonstrate that FPR2 signalling directs cells towards the glycolytic pathway by promoting kinase activity of the bifunctional enzyme PFKFB2 through FGFR/FRS2- and Akt-dependent phosphorylation.

### 3.4. FPR2 signalling prevents the entry of pyruvate in the tricarboxylic acid cycle

**Figure 4.**
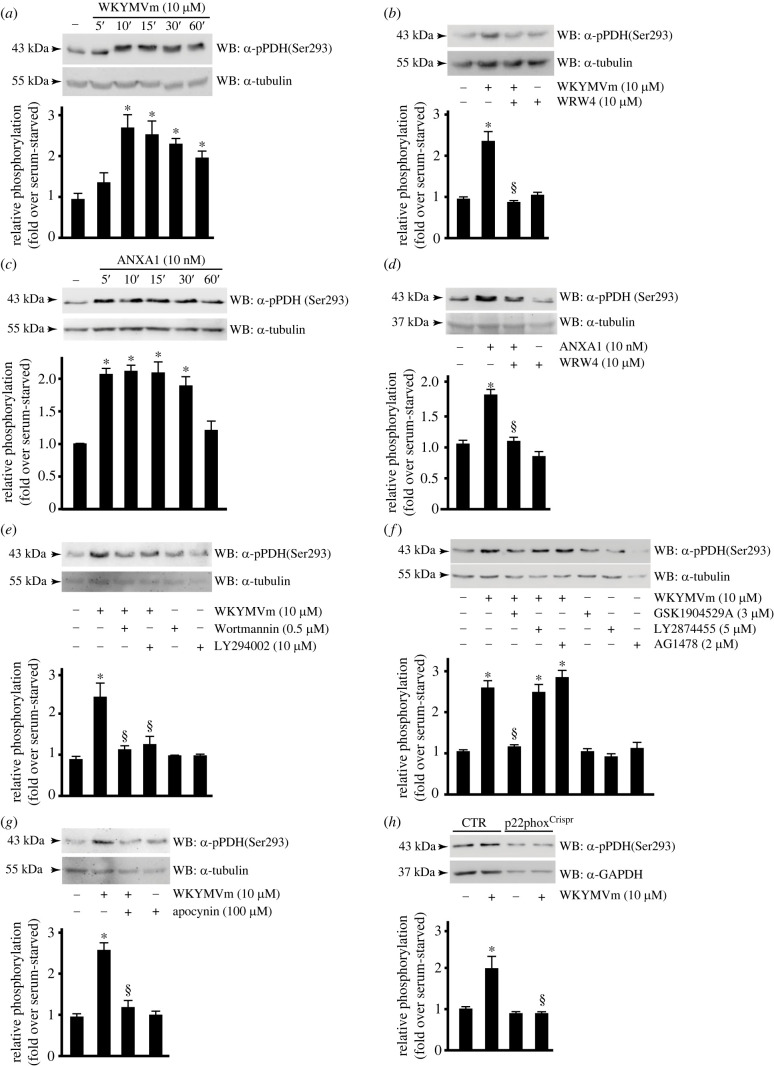
FPR2 signalling prevents pyruvate dehydrogenase activity. (*a*,*c*) FPR2 stimulation induces time-dependent PDH phosphorylation. Serum-deprived CaLu-6 cells were (*a*) stimulated with WKYMVm or (*c*) with ANXA1 for different times. (*b*,*d*) Cells were preincubated with the FPR2 antagonist before stimulation. (*e*) PDH phosphorylation is prevented by PI3K inhibitors. Cells were preincubated with the indicated concentrations of wortmannin or LY294002 before WKYMVm stimulation. (*f*) FPR2-mediated IGF-IR transactivation is required for PDH phosphorylation. Cells were exposed to inhibitors of IGF-IR (GSK1904529A), or FGFR (LY2874455), or EGFR (AG1478) before FPR stimulation. (*g*,*h*) PDH phosphorylation depends on NADPH oxidase activity. (*g*) CaLu-6 cells were preincubated with the indicated concentration of apocynin before stimulation. (*h*) CaLu-6-control^Crispr/Cas9^ cells (CTR) and p22phox^Crispr/Cas9^ (p22phox^Crispr^) cells were serum-starved for 24 h and then stimulated with WKYMVm. Fifty micrograms of whole lysates was resolved on 10% SDS-PAGE and hybridized with an anti-pPDH(Ser293) antibody (α-pPDH(Ser293)). An anti-GAPDH antibody (α-GAPDH) was used as a control for protein loading. Data are representative of three independent experiments. *p < 0.05 compared to unstimulated cells. ^§^*p* < 0.05 compared to WKYMVm-stimulated cells.

PDC is at the centre of aerobic metabolism of carbohydrates. It converts pyruvate into acetyl-CoA and thereby modulates the entry of glucose-derived carbons into the TCA cycle, thus regulating the flow of energy in mammalian cells [88,89]. PDC is composed of three catalytic enzymes and of their respective regulatory proteins [90]. PDC activity is under the control of pyruvate dehydrogenase kinase (PDHK) and pyruvate dehydrogenase phosphatase (PDP), through a reversible phosphorylation–dephosphorylation cycle [91,92]. Pyruvate dehydrogenase is a heterotetrameric enzyme composed of two alpha (PDHA1) and two beta (PDHB1) subunits. PDHA1 is phosphorylated by PDHK1-4 and dephosphorylated by PDP1 and PDP2. The phosphorylation at Ser^293^, Ser^300^ and Ser^232^ residues on PDHA1 decreases PDC activity and contributes to tumour metabolic reprogramming toward glycolysis in hypoxia, by inhibiting acetyl-CoA formation and the entry in the TCA cycle [91,93–95]. We observed that FPR2 stimulation by WKYMVm or ANXA1 induced a comparable PDHA1 phosphorylation kinetics at Ser^293^ residue (figure 4*a*,*c*) that was prevented by preincubation with WRW4 (figure 4*b*,*d*). Similar results were obtained in A549 lung cancer cell line expressing FPR2 [96] (electronic supplementary material, figure S1).

The PI3K signalling pathway regulates glucose metabolism [97,98] and induces, among other things, Thr^346^ phosphorylation and activation of PDHK1 [99]. Active PDHK1 phosphorylates PDHA1 that, in turn, phosphorylates and inactivates PDC. We preincubated cells with wortmannin or LY294002, before WKYMVm stimulation, and observed that this treatment prevents FPR2-induced PDHA1 phosphorylation at Ser^293^ residue (figure 4*e*). Binding of insulin, growth factors, and cytokines to cell surface receptors also triggers PI3K activation. We preincubated cells with specific inhibitors of IGF-IR*β*/IR*β*, FGFR and EGFR and in western blot analysis we observed that only GSK1904529A prevented FPR2-induced PDHA1 Ser^293^ phosphorylation (figure 4*f*), strongly suggesting that it depended on the activation of insulin receptors.

FPRs and activated growth factor receptors increase intracellular ROS generation by activating Nox enzymes or by increasing Nox expression [100,101]. Notably, ROS also modulates Akt activation and MAPK signalling pathways, as well as the activity of several redox-sensitive transcription factors [102,103]. We analysed the role of Nox in PDHA1 regulation and observed that FPR2-induced PDHA1 Ser293 phosphorylation was prevented upon preincubation of cells with the Nox-specific inhibitor apocynin (figure 4*g*) and in the p22phox^Crispr/Cas9^ cells (figure 4*h*) stimulated with WKYMVm.

These results prove that FPR2 signalling induces IGF-IR*β*/IR*β*-, PI3K/Akt- and Nox-dependent inhibition of PDC and, in turn, promotes the aerobic glycolysis pathway for energy production.

### 3.5. WKYMVm stimulation actives lactate dehydrogenase A and enhances lactate production

**Figure 5.**
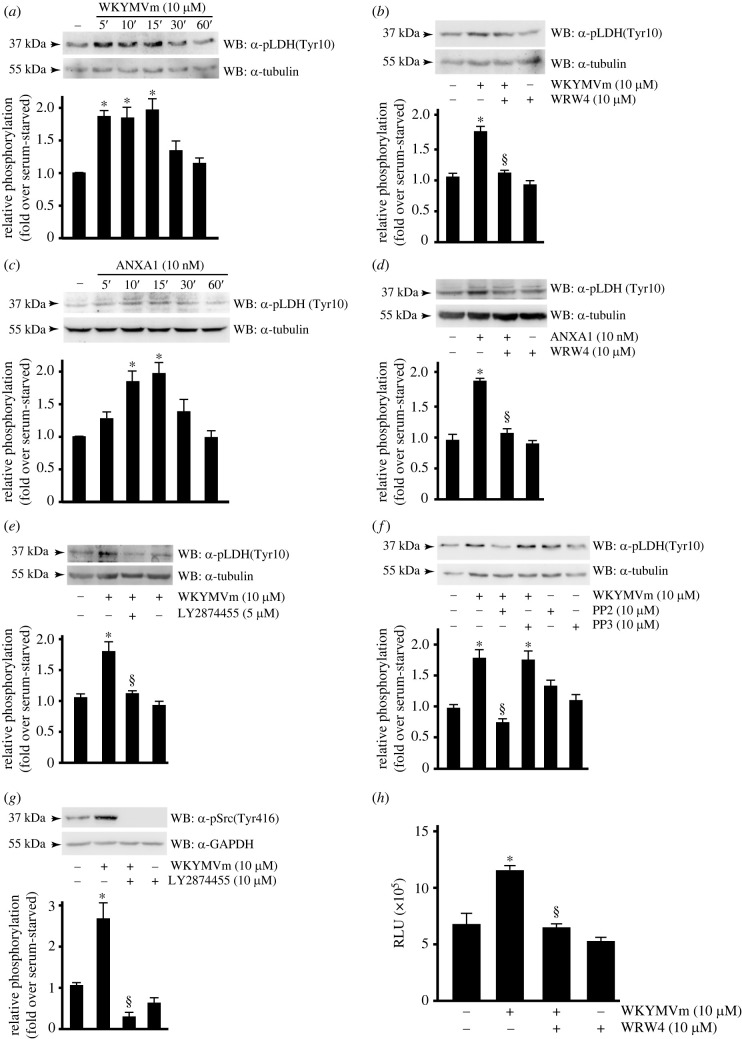
FPR2 stimulation induces LDH activity and an enhanced production of lactate. (*a*–*d*) FPR2 stimulation induces time-dependent LDH phosphorylation. Growth-arrested CaLu-6 cells were stimulated with (*a*) WKYMVm or (*c*) ANXA1 for 5, 10, 15, 30 or 60 min, or (*b*,*d*) preincubated with WRW4. (*e*) LDH activity depends on FPR2-dependent FGFR1 transactivation. Serum-starved cells were preincubated with the FGFR pan-inhibitor LY2874455, at the indicated concentration, before WKYMVm stimulation. (*e*–*g*) FGFR1-recruited Src phosphorylates LDH. (*f*) Cells were preincubated with PP2 or PP3, or (*e*,*g*) with LY2874455, at the indicated concentrations before stimulation. Fifty micrograms of whole lysates was resolved on 10% SDS-PAGE and incubated with (*a*–*f*) an anti-pLDH(Tyr10) antibody (α-pLDH(Tyr10)), or with (*g*) anti-pSrc(Tyr416) (α-pSrc(Tyr416)). An anti-GAPDH antibody (α-GAPDH) was used as a control for protein loading. Data are representative of five independent experiments. (*h*) Representive bar graphs of lactate concentration measured in cell culture media. CaLu-6 cells were serum-starved for 24 h, preincubated with WRW4 and then stimulated with WKYMVm. The media from cell cultures were collected and lactate concentration was measured by using a commercial kit following manufacturer's instructions. Results are the mean of three independent experiments and in each separated experiment every point was analysed in triplicate. *p < 0.05 compared to unstimulated cells. ^§^*p* < 0.05 compared to WKYMVm-stimulated cells.

Lactate dehydrogenase A (LDH-A) catalyses lactate formation from pyruvate and ensures the regeneration of NAD^+^, which is needed as an electron acceptor in glycolysis [104]. In several human cancer cells LDH-A is activated by phosphorylation at Tyr^10^ residue, which correlates with activation of multiple oncogenic tyrosine kinases commonly increased in cancer [105]. By western blot experiments we showed that FPR2 signalling triggered by WKYMVm or ANXA1 induced time-dependent Tyr^10^ LDH-A phosphorylation (figure 5*a*,*c*), that was prevented by the FPR2 antagonist (figure 5*b*,*d*). Similar results were obtained in A549 lung cancer cell line (electronic supplementary material, figure S2).

The oncogenic receptor tyrosine kinase FGFR1 directly phosphorylates LDH-A at Tyr^10^ residue, thus promoting the formation of an active, tetrameric LDH-A complex [105,106]. Since we proved that FPR2 stimulation induces FGFR transactivation (figure 3*d*), we analysed the role of this oncogenic receptor in LDH-A activation and, by immunoblot experiments, we observed that WKYMVm-induced LDH-A phosphorylation at Tyr^10^ residue was prevented by preincubation with the pan-FGFR inhibitor LY2874455 (figure 5*e*). However, other oncogenic tyrosine kinases, such as Src, phosphorylate LDH-A at Tyr^10^ residue [107]. Therefore, we preincubated cells with PP2, an ATP-competitive inhibitor of the Src protein tyrosine kinases family, or with PP3, a negative control for the Src kinase inhibitor PP2, and we observed that Src inhibition prevents LDH-A phosphorylation at Tyr^10^ residue (figure 5*f*). Src can be recruited to active FGFR1 through the adaptor protein FRS2 at the plasma membrane [108,109]. Since Src activity is regulated by phosphorylation on Tyr^416^ residue in the kinase domain, we analysed Src phosphorylation levels in WKYMVm-stimulated cells preincubated or not with the pan-FGFR inhibitor. By western blot analysis with a phospho-specific antibody we observed that LY2874455 prevents Tyr^416^ phosphorylation of Src (figure 5*g*). Furthermore, in line with the FPR2-dependent LDH-A activation, we found that this correlates with an FPR2-dependent increased production of lactate (figure 5*h*). Taken together these results show that in CaLu-6 cells FPR2 signalling triggers FGFR1- and Src-dependent LDH-A activation, thereby promoting lactate production in CaLu-6 cells.

### 3.6. FPR2 stimulation induces HIF-1 and c-Myc activation

**Figure 6.**
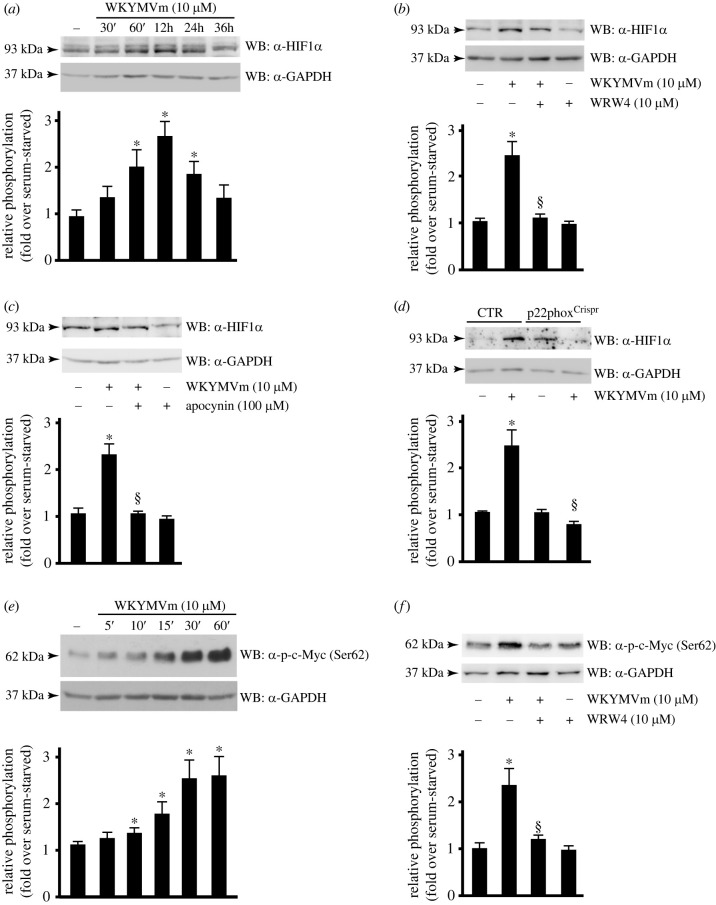
FPR2 activation by WKYMVm induces Nox-dependent HIF-1*α* stabilization and c-Myc phosphorylation. (*a*,*b*) FPR2 signalling triggers time-dependent accumulation of HIF-1*α*. (*a*) Serum-starved CaLu-6 cells were stimulated with WKYMVm for the indicated times, or (*b*) preincubated with WRW4 before stimulation. (*c*,*d*) FPR2-dependent HIF-1*α* stabilization requires Nox2 activity. (*c*) Cells were preincubated with apocynin, before exposure to WKYMVm. (*d*) CaLu-6-control^Crispr/Cas9^ cells (CTR) and p22phox^Crispr/Cas9^ (p22phox^Crispr^) cells were serum-starved for 24 h and then stimulated for 12 h with WKYMVm. (e,*f*) FPR2 signalling triggers time-dependent c-Myc phosphorylation. (*e*) Cells were incubated for increased times with the FPR2 agonist, as indicated, or (*f*) exposed to WRW4 before stimulation. Fifty micrograms of whole lysates was electrophoresed on 10% SDS-PAGE and incubated with (*a*–*d*) an anti-HIF1*α* antibody (α-HIF1*α*), or (*e*,*f*) with an anti-p-c-Myc(Ser62) (α-p-c-Myc(Ser62)). An anti-GAPDH antibody (α-GAPDH) was used as a control for protein loading. Western blot data are representative of five independent experiments. *p < 0.05 compared to unstimulated cells. ^§^*p* < 0.05 compared to WKYMVm-stimulated cells.

LDH-A expression is regulated by c-Myc and hypoxia inducible factor-1 (HIF-1) [110]. These two transcriptional factors cooperate to induce a transcriptional programme for hypoxic adaptation [111], as well as to improve the metabolic needs of cancer cells, by increasing glucose absorption and its conversion to lactate. Hypoxic signalling pathways are implicated in a plethora of physiological processes and they are centrally involved in hyperproliferative disease processes [112]. The central axis of hypoxic signalling is the activation of HIF-1, which consists of an oxygen-regulated HIF-1*α* subunit and a constitutively expressed HIF-1*β* subunit. Under normoxic conditions, HIF-1*α* is hydroxylated on two proline residues by prolyl hydroxylases, leading to its rapid proteasomal degradation. By contrast, hypoxic conditions inhibit HIF-1*α* degradation leading to its stabilization and nuclear translocation [113]. In the nucleus, HIF-1*α* dimerizes with HIF-1*β* and binds to cis-acting hypoxia response elements (HREs) in several target genes, including those involved in glucose uptake, glycolytic enzyme synthesis, lactate generation and secretion [114]. Therefore, we first evaluated the ability of FPR2 to induce HIF-1*α* stabilization and, in immunoblot experiments performed on whole protein extracts of CaLu-6 cells, we observed a time-dependent accumulation of this protein (figure 6*a*), which was prevented by WRW4 (figure 6*b*). Nox-dependent ROS generation is involved in hypoxic signalling in primary lung cells and, in turn, in HIF-1*α* stabilization [112]. In agreement, we observed that Nox inhibition by apocynin (figure 6*c*) or by CRISPR/Cas9-based p22^phox^ editing (figure 6*d*) prevents HIF-1*α* accumulation in FPR2-stimulated CaLu-6 cells.

FPR2 localizes also in nuclear fractions of CaLu-6 and AGS cells and nuclear FPR2 activation prompts a decreased G*α*i-FPR2 association and triggers ERKs, c-Jun and c-Myc activation [26]. In response to a growth-stimulatory signal, c-Myc protein is phosphorylated at Ser^62^ residue, which results in its stabilization [115]. Interestingly, by western blot analysis performed in WKYMVm-stimulated CaLu-6 cells with an anti-Myc(pSer62) antibody, we detected a time-dependent increase of Myc phosphorylation (figure 6*e*), which was prevented by FPR2 antagonist pretreatment (figure 6*f*).

These results demonstrate that FPR2 signalling controls HIF-1 and c-Myc activation, which are involved in the transcriptional regulation of genes involved in the metabolism of glucose.

### 3.7. FPR2 stimulation improves energetic metabolism of CaLu-6 cells

**Figure 7.**
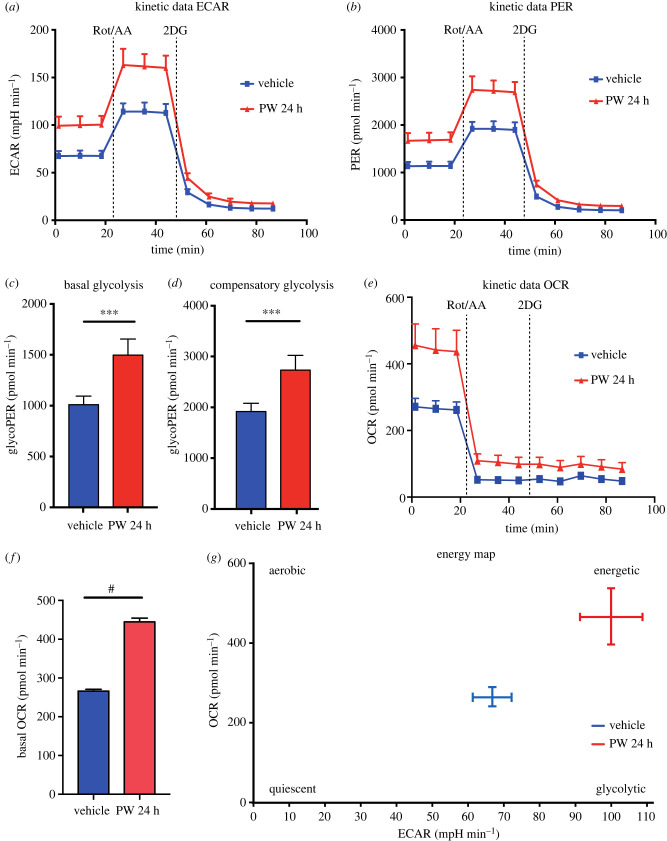
Seahorse analysis of WKYMVm-stimulated CaLu-6 cells. Extracellular acidification rate (ECAR) (*a*) and proton efflux rate (PER) (*b*) were measured in Calu-6 cells treated for 24 h with 10 µM WKYMVm (red line) or vehicle (blue line). Basal ECAR and PER measurements were followed by sequential treatment (dotted vertical lines) with rotenone plus antimycin A (Rot/AA) and 2-deoxyglucose (2DG). Bar graphs represent (*c*) basal and (*d*) compensatory PER produced from glycolysis (glycoPER). (*e*,*f*) Basal oxygen consumption rate (OCR) was measured in Calu-6 cells treated with 10 µM WKYMVm (red line) or vehicle (blue line) for 24 h. (*g*) Metabolic profile of CaLu-6 cells exposed to 10 µM WKYMVm (red) or vehicle (blue) for 24 h. ***p < 0.001 and ****p < 0.0001 compared to unstimulated cells.

We further evaluated the effect of FPR2 stimulation on glucose metabolism in lung cancer CaLu-6 cells by using Seahorse XF glycolytic rate assay. This assay provides accurate measurements of glycolytic rates for basal conditions and compensatory glycolysis following mitochondrial inhibition. The calculated rates account for contribution of CO_2_ to extracellular acidification derived from mitochondrial/TCA cycle activity and are directly comparable to lactate accumulation data. Firstly, we measured the real time extracellular acidification rate (ECAR) in serum-starved cells stimulated or not with WKYMVm for 24 h. Kinetic data showed a significant increase of the ECAR in FPR2-stimulated cells (figure 7*a*). In addition, the proton efflux rate (PER) value provides a more accurate measurement of extracellular acidification (pmol H^+^ min^−1^), by calculating the total proton efflux derived from glycolytic and mitochondrial acidification. Consistent with ECAR, PER was significantly enhanced upon WKYMVm exposure in CaLu-6 cells compared to unstimulated cells (figure 7*b*). Furthermore, inhibition of mitochondrial respiration by rotenone and antimycin A (Rot/AA) was used to calculate the glycolytic proton efflux rate (glycoPER), thus estimating the proton efflux derived from glycolysis. Our results showed that FPR2 stimulation, measured after blockade of mitochondrial electron transport chain, significantly upregulated glycoPER in both basal (figure 7*c*) and compensatory glycolysis (figure 7*d*). In addition, WKYMVm stimulation significantly increased mitochondrial basal respiration compared to untreated CaLu-6 cells, as suggested by oxygen consumption rate (OCR) measurement (figure 7*e*,*f*). Previously we demonstrated that FPR2 stimulation significantly improves the expression of the glutamine transporter ASCT2, which correlates with an increase of glutamine uptake [45]. Glutaminase converts glutamine in glutamate that is transaminated in alpha-ketoglutarate. This fuels the TCA to generate ATP and citrate contributing to mitochondrial respiration and thus to an increase of OCR. Notably, FPR2-stimulated cells showed significant changes in both OCR and ECAR compared to unstimulated cells, suggesting a switch towards a more energetic phenotype (figure 7*g*).

Taken together, these data clearly demonstrate that FPR2 stimulation enhances energetic metabolism of Calu-6 cells.

## 4. Discussion

By using a metabolomic approach, we have analysed metabolic pathways activated in FPR2-stimulated CaLu-6 cells, a human lung cancer cell line. Metabolic data reveal that FPR2 stimulation increases cellular concentration of metabolites involved in glucose metabolism, such as glucose 6P, F1,6BP, GA3P and lactate. We prove that FPR2 stimulation enhances glucose uptake in a time-dependent manner by increasing GLUT4 cellular membrane localization through insulin receptor-dependent PI3K/Akt signalling cascade. FPR1 stimulation, another member of the FPR family expressed in a range of tissues and cell types [116], also enhances glucose uptake and GLUT4 translocation via Akt activation [117]. Furthermore, the FPR1 agonist formyl-methionyl-leucyl-phenilalanine (fMLP) peptide induces GLUT1 and GLUT5 membrane translocation in human monocytes [118] and stimulates 2-deoxyglucose uptake in macrophage in association with an increase of GLUT3 on the membrane [119]. GLUT4 is the insulin-regulated member of transmembrane glucose transporter family and consistently we show that WKYMVm stimulation triggers FPR2- and Nox2-dependent IGF-IR*β*/IR*β* trans-phosphorylation. GPCRs and TKRs are not to be only considered as distinct signalling units; indeed GPCR-mediated TKR transactivation is a proven molecular mechanism able to increase the number and range of cellular signalling networks. IGF-IR is transactivated by GABA_B_, thrombin, metabotropic glutamate, neurotensin and angiotensin II (AngII) type receptors [120–124]. In this paper, we provide the first demonstration that FPR2 functionally transactivates IGF-IR in a human cancer cell line.

We prove that FPR2 signalling directs cells towards the glycolytic pathway by promoting Akt- and FGFR-dependent kinase activity of the bifunctional enzyme PFKFB2. Several GPCRs form heterocomplexes with FGFRs and control the cell fate [125–133]. Our data reveal for the first time in epithelial cancer cells a cross talk between FPR2 and FGFR1, as well as the activation of the scaffold phosphoprotein FSR2, which acts as a docking protein downstream to phosphorylated FGFR1.

Pyruvate arising from glycolysis can be converted in acetyl-CoA by an oxidative decarboxylation catalysed by PDH, or in lactate by an oxidoreduction reaction catalysed by LDH. In cancer cells the production of lactate and H^+^ ions plays crucial roles in: (i) synthesis of NAD^+^ necessary to sustain the increased rate of glycolysis; (ii) acidification of the tumour microenvironment, thus reducing the viability of normal cells and favouring the infiltration of neoplastic cells [134]; and (iii) binding to specific receptors on target cells, such as GPR81, thus activating intracellular signalling cascades, lactate uptake, mitochondrial metabolism, angiogenesis and tumour growth [135–137]. We demonstrate that FPR2 signalling triggers PDHK1-mediated PDHA1 phosphorylation at Ser^293^. Therefore, by suppressing the oxidative decarboxylation of pyruvate, phosphorylated PDHK1 shuts off oxidative phosphorylation, maintains tumour cell proliferation in severe hypoxia conditions, and switches cancer metabolism towards glycolysis. We also reveal an increase of LDH-A activity that is involved in lactate production and that significantly contributes to the Warburg effect [138,139]. Cancer cells reprogramme their metabolism to support survival, growth and proliferation, and they synthesize large amounts of lactate independently of the oxygen availability. Since the oxidation of glucose to lactate generates 2 ATPs per molecule of glucose, whereas oxidation of pyruvate in TCA and oxidative phosphorylation generate up to 36 ATPs, the Warburg effect has been proposed as a mechanism to support the biosynthetic requirements of cancer cells. In fact, carbon atoms derived from the increased glucose consumption can be used for anabolic processes needed to support cell proliferation, such as de novo synthesis of nucleotides, lipids, and proteins [140–143]. This implies that cancer cells are in greater need of reducing equivalents in the form of NADPH, which is necessary for reductive biosynthesis. Increased glucose uptake allows an enhanced synthesis of NADPH in the oxidative branch of PPP which also provides ribose-5P for the synthesis of nucleotides. Accordingly, in our metabolomic analysis we observed an increase of NADPH production via PPP and the activation of the multifunctional enzyme CAD that participates in the three initial speed-limiting steps of the de novo synthesis of pyrimidine nucleotides in mammals [45]. The regeneration of NAD^+^ from NADH in the reaction catalysed by LDH represents another mechanism that accounts for the biosynthetic function of the Warburg effect. In this scenario NADH is consumed to regenerate NAD^+^, to keep glycolysis active in cancer cells and to allow the biosynthesis of serine from 3-phosphoglycerate. Serine is required for many biosynthetic and signalling pathways and provides a carbon unit into the folate-dependent biosynthesis of purine nucleotides [144].

We prove that FPR2 signalling induces HIF-1 stabilization and c-Myc activation. Interestingly, these two transcriptional factors cooperate to regulate LDH-A expression and to activate hexokinase 2 and PDK1, resulting in enhanced conversion of glucose to lactate [145]. HIF-1 is also a determinant for GLUT4-mediated glucose uptake [146].

Nox2-dependent ROS generation plays also a crucial role in the molecular mechanisms that we herein describe. In fact, we show that ROS cellular levels regulate (i) GLUT4 membrane localization; (ii) FPR2-mediated IGF-IR*β*/IR*β* transactivation; (iii) PDH phosphorylation; and (iv) HIF1*α* stabilization. Accordingly, in skeletal muscle fibres, Nox2 regulates glucose transport capacity through GLUT4 and AngII-mediated IGF-1R transactivation [147,148]. Furthermore, some evidence suggests that ROS inhibition prevents PDH phosphorylation [149] and that ROS may activate PDKs [150]. Nox-derived ROS can also enhance HIF activation [151,152]. In fact, the increase in ROS generation observed in cells overexpressing Nox1 is associated with the activation of HIF-1-dependent target gene expression [153], and Nox4 activation by thrombin increases HIF-2*α* protein levels [154]. Interestingly, Nox4 is a transcriptional target of HIF-1*α* [155]. However, further studies in models of lung cancer should be performed in order to extend the knowledge on the role of FPR2 in metabolic reprogramming.

## 5. Conclusion

**Figure 8.**
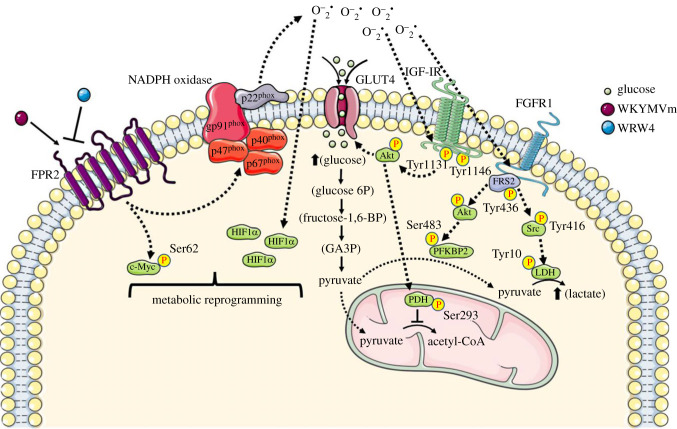
Integration between signalling pathways triggered by FPR2 and glucose metabolism. FPR2 stimulation by WKYMVm induces NADPH oxidase-dependent ROS generation, which is involved in IGF-IR and FGFR transactivation. IGF-IR stimulates cellular glucose uptake by inducing Akt-dependent translocation of GLUT4 to the plasma membranes. FPR2 signalling directs glucose towards the glycolytic pathway by promoting kinase activity of the bifunctional enzyme PFKFB2 through FGFR/FRS2/Akt-dependent phosphorylation. Intracellular pathways triggered by FPR2 also induce IGF-IR/Akt-dependent inhibition of PDH and, in turn, promote aerobic glycolysis pathway for energy production. Src, activated by FGFR/FRS2 cascade, phosphorylates LDH with the consequent enhanced production of lactate. ROS induce Nox-dependent HIF-1*α* stabilization and c-Myc phosphorylation which cooperate to regulate LDH expression.

The emerging view of metabolic regulation in cancer cells is that signal transduction networks participate in a substantial reorganization of metabolic activities. Since Warburg's early observations, much information on glucose metabolism in cancer cells has been understood, but the integration between signalling pathways and cellular metabolism is still unclear. This study provides new insights into the molecular mechanisms by which FPR2-induced/TKR signalling and Nox2-dependent ROS generation regulate glucose metabolism in CaLu-6 cancer cells. FPR2 stimulation triggers intracellular signalling cascades that induce TKR transactivation, insulin-dependent glucose uptake, the activation of regulatory glycolytic enzymes, the promotion of aerobic glycolysis for energy production, instead of mitochondrial oxidative phosphorylation, and both an enhanced LDH activity and lactate production (figure 8). Therefore, FPR2 signalling and Nox2 regulatory subunits are promising therapeutic targets to be explored for the treatment of human cancers.

## Data Availability

The data are provided in electronic supplementary material [156].
